# Poverty, partner discord, and divergent accounts; a mixed methods account of births before arrival to health facilities in Morogoro Region, Tanzania

**DOI:** 10.1186/s12884-016-1058-x

**Published:** 2016-09-27

**Authors:** Shannon A. McMahon, Rachel P. Chase, Peter J. Winch, Joy J. Chebet, Giulia V. R. Besana, Idda Mosha, Zaina Sheweji, Caitlin E. Kennedy

**Affiliations:** 1 Department of International Health, Johns Hopkins Bloomberg School of Public Health, 615 N. Wolfe Street, Baltimore, MD 21205-2179 USA; 2 Institute of Public Health, Heidelberg University, Im Neuenheimer Feld 324, D-69120 Heidelberg, Germany; 3 Monitoring & Evaluation Office, Jhpiego, PO Box 9170, Dar es Salaam, Tanzania; 4 School of Public Health and Social Sciences, Muhimbili University of Health and Allied Sciences, P.O. Box 65015, Dar es Salaam, Tanzania; 5 Independent Consultant, Moshi, Tanzania; 6 Independent Consultant, Dar es Salaam, Tanzania

**Keywords:** Tanzania, Birth before arrival, Maternal health, Newborn health, Spouses, Poverty, Social class, Delivery

## Abstract

**Background:**

Births before arrival (BBA) to health care facilities are associated with higher rates of perinatal morbidity and mortality compared to facility deliveries or planned home births. Research on such births has been conducted in several high-income countries, but there are almost no studies from low-income settings where a majority of maternal and newborn deaths occur.

**Methods:**

Drawing on a household survey of women and in-depth interviews with women and their partners, we examined the experience of BBA in rural districts of Morogoro Region, Tanzania.

**Results:**

Among survey respondents, 59 births (4 %) were classified as BBAs. Most of these births occurred in the presence of a family member (47 %) or traditional birth attendant (24 %). Low socioeconomic status was the strongest predictor of BBA. After controlling for wealth via matching, high parity and a low number of antenatal care (ANC) visits retained statistical significance. While these variables are useful indicators of which women are at greater risk of BBA, their predictive power is limited in a context where many women are poor, multiparous, and make multiple ANC visits. In qualitative interviews, stories of BBAs included themes of partner disagreement regarding when to depart for facilities and financial or logistical constraints that underpinned departure delays. Women described wanting to depart earlier to facilities than partners.

**Conclusion:**

As efforts continue to promote facility birth, we highlight the financial demands associated with facility delivery and the potential for these demands to place women at a heightened risk for BBAs.

**Electronic supplementary material:**

The online version of this article (doi:10.1186/s12884-016-1058-x) contains supplementary material, which is available to authorized users.

## Background

Deliveries that occur before arrival at a health care facility (birth before arrival or BBA) – also called accidental, unplanned or out-of-hospital births or deliveries – are associated with higher rates of maternal and neonatal morbidity and mortality compared to facility deliveries or planned home births [1–3]. Maternal outcomes highlighted in a recent review include increased complications and higher morbidity including more frequent tearing, increased blood loss and increased risk of longer third stage of labour [2]. Neonatal complications associated with BBAs include low body temperature and low blood glucose [4] as well as low birth weight [1, 2, 5, 6]. Across several studies, neonatal mortality following BBAs has been reported as six to 11 times greater than hospital births [2], and BBAs constitute an outsized proportion of death given the relative rarity of a BBA event [7]. Only one study, from Australia, has qualitatively examined BBAs; it concluded that a BBA leads to heightened feelings of anxiety and fear among mothers [8].

While research on BBAs has been conducted in several high-income countries, there are almost no studies from low-income settings where a majority of maternal and newborn deaths occur. BBAs are uncommon globally with reported rates across high-income countries ranging from 0.08 % [5] to 1.99 % [4]. Temporal trends reveal a rise in BBAs in some wealthy nations [9], including a doubling of the incidence in Finland between the 1970s and 1990s [10] and Ireland between 2005 and 2009 [11], but staying below 0.5 % in these cases. These increases have been attributed to closures of remote maternity wards and subsequent transport barriers for rural women [1, 10], a larger migrant population [11] and delayed departure to facilities among labouring women [3] including those living near a facility [6].

In high-income settings, the most widely agreed upon predisposing factor for BBAs is poor antenatal care attendance [3, 4, 7, 10, 12–16], although this trend has not been universal [6]. Groups found to be at increased risk for BBA include young women [14], single women [1, 7, 12], women from disadvantaged socio-economic backgrounds [11, 17], young primigravida women [18], as well as older, multigravida women [18]. Two distinct groups of women described in the literature who appear predisposed to BBA are: women who are older, multiparous and with shorter labors [3, 6, 10, 12, 18, 19], or women, typically of a young age, who are attempting to conceal their pregnancy [1, 7, 12]. Quantitative research on trends and patterns in BBAs has been conducted in Australia [2], England [1, 6, 12], Finland [10], France [14, 16], Hong Kong [17], Ireland [7, 11], Israel [20], Italy [5], Japan [13], Scotland [3] and the United States [4, 21]. A majority of these studies reported on BBAs that occurred in the presence of a woman’s partner, without a medical professional and within a woman’s home, but also in ambulances, emergency rooms, hospital entrances, taxis, parking lots and public bathrooms.

To our knowledge, one study has addressed BBAs in Africa. In a prospective one-to-one matching study in South Africa, BBAs were associated with distance to obstetric wards, parity, complications during pregnancy, shorter duration of labor and a previous home delivery [22]. In a study from Zambia that compared women’s perceptions of home and facility births, respondents described how concerns about giving birth en route contributed to a preference for home delivery [23].

Limitations within the existing BBA literature confine our understanding of the issue. First, there is limited qualitative literature on the experience of BBAs [8]. Second, there is a paucity of literature on BBAs in low-income countries. Finally, we are not aware of BBA literature that incorporates perspectives of male partners, although the role of men in deciding whether or when to seek care outside the home has emerged as crucial in the maternal health literature [24], including literature from Tanzania [25]. In this paper, we aim to highlight risk factors for a BBA in a low-income setting and to describe how local reproductive health norms and practices, including how couples define and negotiate each partner’s role in careseeking for birth, can shape circumstances surrounding a BBA.

### Study location

In Tanzania, home and facility births occur in relatively even proportions. As of 2010, 50.1 % of births occur in a facility, which approximates the rate of facility births reported in 1991–2 [26, 27]. Maternal and neonatal mortality are high with 454 maternal deaths and 2586 neonatal deaths per 100,000 live births; the total fertility rate is 5.3 [26]. One in 38 women will die due to maternal causes in her lifetime [28]. For every 1000 births, four to five women die from pregnancy-related causes and 26 neonates die [26]. Tanzania is among ten countries that account for a majority of the world’s “first-day deaths,” or death on the first day of life [28]. In Morogoro region, nearly one quarter of women have no education, 34.3 % have had some primary education and 32.8 % have completed primary school [26]. While most women can read a whole sentence, 26 % of women cannot read at all [26]. More than 60 % of men and women are engaged in agriculture as their primary occupation [26]. In the Eastern Zone, which encompasses Morogoro region, the infant mortality rate is 70 deaths per 1000 live births [26]. Maternal mortality estimates are not available at the regional level in the Demographic and Health Survey [26].

## Methods

This mixed methods, cross sectional study was conducted as part of a larger program evaluation of a maternal and neonatal health program designed to encourage uptake of facility-based services related to pregnancy and childbirth in Morogoro Region. Drawing on a household survey and in-depth interviews conducted in Morogoro Region, Tanzania, this study presents quantitative and qualitative data on BBAs across four rural districts (Morogoro Rural, Kilosa, Ulanga, and Mvomero District Council). Women and their partners were eligible to participate in the study if the family had experienced a birth within the preceding 14 months (a cutoff set with the intention of reducing recall bias). We define a BBA as the occurrence of a birth prior to arrival at a health facility when, according to respondents, that birth was intended to occur in a health facility. This is consistent with a BBA definition employed by Tanzanian health facilities for record-keeping, although in some facilities practitioners also require that a mother present physical evidence of a recent birth (such as a placenta or other birth remnants), which is considered a means to verify place-of-delivery intent (the authors can only speculate on how this practice serves to verify intent).

### Study design

#### Training

Research managers trained teams of five qualitative and 16 quantitative research assistants on research methods, ethics and various facets of careseeking for maternal and neonatal health during antenatal, intrapartum and postpartum periods. Trainings were followed by pilot testing and tool revision among respondents who fit the study’s eligibility criteria (detailed below) and who lived in or near Dar es Salaam (where trainings took place). Research assistants were college-educated Tanzanians, with a relatively even breakdown of males and females trained as teachers, health practitioners and social sciences or public health graduate students.

#### Mixed methods design

This study employed a mixed-methods convergent parallel design, wherein qualitative and quantitative data were collected during approximately the same time period, and the aim was to compare and contrast findings across the two strands of data [29]. Qualitative data analysis preceded and informed quantitative data analysis. Further details of analysis are presented below.

#### Quantitative design and sampling

The household survey (see Additional file 1) was designed to be regionally representative of rural Morogoro and self-weighting via a multistage cluster sampling survey. Data were collected from August to November in 2011. Sixty clusters were identified via probability proportional to size (PPS) sampling methods and 30–35 women were surveyed in each cluster. In each cluster, the survey team visited all households to identify eligible women. If a household had more than one eligible woman, the interviewer compiled a list of the eligible women in the household and randomly selected one from the list. Further details of our quantitative design including details of the sampling frame and clusters have been outlined in related publications [30, 31].

#### Quantitative analysis

Quantitative analyses were conducted with Stata (Version 13.1, College Station, TX). Data points for 59 births before arrival and 1267 facility births were analyzed. For this analysis, a birth was categorized as a BBA if a woman responded to the question “Where did your delivery occur?” by stating that the delivery took place “On the way to a facility” or “In the home of a stranger while on the way to a facility”. A birth was categorized as a facility birth if a woman stated that a birth occurred in any type of hospital (regional, district, or local) or health center or dispensary. Due to the rarity of the outcome, few parameters could be estimated with logistic regression, and the effects of multicollinearity could have been easily amplified [32]. To avoid estimation errors, we performed a series of analyses. In each phase of the analysis, we first estimated the effect of the most prominent predictor, then matched cases and controls on that predictor so that we could assess the effect of less prominent predictors in the next phase while controlling for previously identified predictors via matching. In this way, we matched on key variables and estimated effects on a limited number of remaining variables. To determine key variables for matching, we conducted logistic regressions to assess for risk factors associated with the outcome variable (BBA). Variables included age, parity, education and wealth, where wealth was calculated using a principal components analysis of household assets [33]. We then used linear regression to assess the relationship of the strongest predictors of BBAs with all other predictors. Finally, we assessed candidate key variables for matching potential, that is, whether enough matches existed in the data to use a key variable as a matching variable. For example, socio-economic status was considered a key variable because it was the strongest predictor of BBA, it was highly correlated with other predictors, and it offered multiple matches among the 1267 facility births and the 59 BBAs. Our identification of key variables was also informed by existing BBA and maternal health literature, which highlights the importance of age at delivery, age at first pregnancy, parity, wealth, number of ANC visits, maternal education and partner education. To match data points, we performed the coarsened exact matching procedure using the Stata command cem, which maximizes the number of matches while retaining a meaningful level of precision in matches [34]. We report unadjusted odds ratios for each predictor variable. At each stage of matching, unadjusted odds ratios were reported for all remaining variables, and those with *p*-values <0.10 were included in a multiple logistic regression model from which adjusted odds ratios were reported.

#### Qualitative design and sampling

In-depth interviews (see Additional file 2) with women and partners who experienced BBAs were selected from a larger study on careseeking during pregnancy and childbirth among women (*n* = 49) and their partners (*n* = 27) living near (<3 km) and far (≥3 km) from facilities. Women were neither more likely to be selected nor ineligible for the qualitative portion of the study if they were selected for the quantitative survey. Data were collected in July and August of 2011. Researchers identified women for the larger study through engagement with village health committees and canvassing villages to invite eligible mothers to participate. Women and partners for the larger study were not recruited based on their experience of a BBA. The BBA experience was identified as salient during data collection and concurrent qualitative analysis, leading to increased probing regarding BBA during later interviews. All interviews indicating a BBA experience were selected for this analysis, yielding 13 interviews (including six follow-up interviews) with four women and three partners, with one woman per district. Further details of our qualitative design and sampling have been outlined in related publications [35].

Data collectors interviewed all respondents one-on-one, in a private location of their choosing. The interview included open-ended questions such as “Could you please walk me through your experience from the moment you sensed the baby was going to be born?” and probes such as “Please tell me more about that”. Data collectors were instructed to respect respondent autonomy - particularly in the event that sensitivities or uneasiness emerged during interviews. In the event of inconsistencies comparing women’s versus their husbands’ accounts of the birth experience, interviewers were careful not to highlight or probe on these inconsistencies with either party. A field supervisor led daily debriefing sessions with the qualitative team throughout data collection to triangulate findings, strengthen probing, build field notes, identify topics to address in future interviews and develop themes for a codebook that would later be applied to transcripts.

#### Qualitative analysis

Qualitative analysis drew upon 13 in-depth interviews (IDI), including six follow-up interviews, from four husband-wife pairs who experienced a BBA. In this study, follow-up interviews were conducted to clarify points that were considered unclear or insufficiently discussed in the analysis of the initial interview. Interviews with one partner had to be repeatedly cancelled due to his intoxication (from alcohol). All interviews were recorded, transcribed and quality controlled by bilingual researchers to ensure that the content of the recorded interview was reflected in the transcript. A case study approach was used to analyze and present the qualitative data [36–38]. Cases were first assembled from raw case data drawn from interviews with women and their partners. In line with Patton 2002, narratives were written in a manner intended to present a fluid and coherent description of the BBA in a chronological fashion [36]. Case study researchers emphasize that “the analyst’s first and foremost responsibility consists of doing justice to each individual case” [36]. Due to space limitations, we could not include narratives as holistic entities within this article; however narratives can be read as supplementary data (see Additional file 3). Results of a cross-case analysis, which was conducted to inductively identify themes across narratives, are detailed in the results section.

## Results

### Quantitative results

#### Household survey data

Table 1 provides characteristics of BBA (*n* = 59) and facility (*n* = 1267) births as well as respondent characteristics used in the matched pairs analysis. During delivery, most women who experienced a BBA were attended by a traditional birth attendant (TBA) (27 %) or a family member (46 %). A majority of newborns born en route cried at birth (86 %), and most were still alive at the time of the survey (93 %). Table 2 provides respondent characteristics that were used in the matched pairs’ analysis. Women who experienced a BBA were generally married (81 %), lived in male-partner headed households (73 %), had four or more children (51 %), and had their first child by age 19 (84 %). A majority of women who experienced a BBA (51 %) and their husbands (88 %) had completed primary education, but 37 % reported having no education. All 59 women who experienced a BBA reported at least one ANC visit and most (63 %) reported attending four or more visits.

**Table 1:** Sample description from household survey with *N* = 59 births before arrival and *N* = 1267 facility births

Indicator (BBA n; facility delivery n)	Birth before arrival n (%)	Birth in facility n (%)
	59	1267
Characteristics of delivery		
Who assisted at birth (59; 1267) (could select more than one)		
Brother/sister/friend/neighbor	27 (46 %)	36 (3 %)
Traditional Birth Attendant	16 (27 %)	17 (1 %)
Another person (unstated)	13 (22 %)	18 (1 %)
Community Health volunteer	3 (5 %)	34 (3 %)
Traditional Healer	3 (5 %)	1 (0 %)
Health worker at dispensary	2 (3 %)	562 (44 %)
Health worker at health center	0 (0 %)	363 (29 %)
Health worker at hospital	0 (0 %)	293 (23 %)
None of the above	5 (8 %)	95 (7 %)
Newborn cry at birth (59; 1267)		
Yes	51 (86 %)	1068 (84 %)
No	4 (7 %)	151 (12 %)
No response	4 (7 %)	48 (4 %)
Newborn still alive (59; 1267)		
Yes	55 (93 %)	1218 (96 %)
No	2 (3 %)	34 (3 %)
No response	2 (3 %)	15 (1 %)
Characteristics of respondent		
Age (59; 1266)		
Under 19 years	6 (10 %)	203 (16 %)
20 to 33 years	40 (68 %)	858 (68 %)
More than 34 years	13 (22 %)	205 (16 %)
Age at first pregnancy (59; 1258)		
Under 16 years	12 (20 %)	140 (11 %)
16 to 19 years	38 (64 %)	755 (60 %)
More than 19 years	9 (15 %)	363 (29 %)
Parity (59; 1263)		
1	4 (7 %)	312 (25 %)
2–3	25 (42 %)	496 (39 %)
4+	30 (51 %)	455 (36 %)
Woman’s education (59; 1246)		
None	22 (37 %)	280 (22 %)
Some Primary	6 (10 %)	135 (11 %)
Primary Complete	30 (51 %)	749 (60 %)
Secondary or Higher	1 (2 %)	82 (7 %)
Partner’s education (42; 951)		
None	0 (0 %)	20 (2 %)
Some Primary	4 (10 %)	68 (7 %)
Primary Complete	37 (88 %)	757 (80 %)
Secondary or Higher	1 (2 %)	106 (11 %)
Asset index^a^ quintile (59; 1267)		
Lowest	18 (31 %)	231 (18 %)
Second	20 (34 %)	179 (14 %)
Middle	11 (19 %)	232 (18 %)
Fourth	7 (12 %)	291 (23 %)
Highest	3 (5 %)	334 (26 %)
Number of ANC visits (57; 1243)		
1	3 (5 %)	13 (1 %)
2	6 (11 %)	69 (6 %)
3	12 (21 %)	294 (24 %)
4 or more	36 (63 %)	867 (70 %)
Relationship to head of household (59;1261)		
Self	7 (12 %)	111 (9 %)
Wife	43 (73 %)	892 (71 %)
Daughter	5 (8 %)	155 (12 %)
Other	4 (7 %)	103 (8 %)
Marital status (59; 1266)		
Married	48 (81 %)	1017 (80 %)
Divorced, Separated, or Widowed	5 (8 %)	97 (8 %)
Never Married	6 (10 %)	152 (12 %)

^a^Asset index estimated using the first component of principal components analysis of asset ownership

**Table 2:** Factors associated with birth before arrival at a facility based on household survey in rural Tanzania*

	No matching unadjusted odds ratio (CI)	No matching adjusted odds ratio (CI)	Matching^a^unadjusted odds ratio (CI)	Matching^a^adjusted odds ratio (CI)	Matching^b^unadjusted odds ratio (CI)	Matching^b^adjusted odds ratio (CI)
	Column 1	Column 2	Column 3	Column 4	Column 5	Column 6
Age	*p* = 0.228	*p* = 0.931	*p* = 0.307	NA	*p* = 0.217	NA
Under 16 years	1 (ref)	1 (ref)	1 (ref)		1 (ref)	
16 to 19 years	1.58 (0.67, 3.71)	1.07 (0.31, 3.70)	1.77 (0.76, 4.10)		1.82 (0.80, 4.17)	
More than 19 years	2.15 (0.89, 5.19)	1.28 (0.32, 5.22)	1.96 (0.82, 4.72)		2.19 (0.90, 5.33)	
Age at first pregnancy	*p* = 0.008	*p* = 0.074	*p* = 0.040	*p* = 0.063	NA	NA
Under 16 years	1 (ref)	1 (ref)	1 (ref)	1 (ref)		
16 to 19 years	0.59 (0.27, 1.26)	0.79 (0.33, 1.86)	0.65 (0.30, 1.38)	0.68 (0.31, 1.46)		
More than 19 years	0.29 (0.13, 0.65)	0.48 (0.18, 1.27)	0.35 (0.16, 0.80)	0.38 (0.16, 0.87)		
Parity	*p* = 0.009	*p* = 0.108	*p* = 0.019	*p* = 0.023	*p* = 0.027	*p* = 0.024
1	1 (ref)	1 (ref)	1 (ref)	1 (ref)	1 (ref)	1
2–3	3.93 (1.36, 11.36)	4.79 (0.94, 24.5)	4.01 (1.41, 11.4)	3.88 (1.33, 11.3)	3.98 (1.39, 11.4)	4.05 (1.37, 11.9)
4+	5.14 (1.82, 14.56)	4.65 (0.74, 29.1)	4.22 (1.52, 11.8)	4.47 (1.53, 13.1)	3.90 (1.40, 10.8)	4.30 (1.49, 12.4)
Woman's education	*p* = 0.108	*p* = 0.896	*p* = 0.735	NA	*p* = 0.661	NA
None	1 (ref)	1 (ref)	1 (ref)		1 (ref)	
Some Primary	0.57 (0.19, 1.68)	0.87 (0.24, 3.11)	0.71 (0.23, 2.18)		0.51 (0.14, 1.84)	
Primary Complete	0.51 (0.27, 0.96)	0.91 (0.37, 2.26)	0.76 (0.40, 1.45)		0.82 (0.42, 1.60)	
Secondary or Higher	0.16 (0.02, 1.22)	0.87 (0.10, 7.55)	0.37 (0.04, 3.07)		0.39 (0.05, 3.19)	
Partner's education	*p* = 0.425	*p* = 0.583	*p* = 0.773	NA	*p* = 0.794	NA
None/Some Primary	1 (ref)	1 (ref)	1 (ref)		1 (ref)	
Primary Complete	1.08 (0.31, 3.7)	2.59 (0.54, 12.4)	1.43 (0.40, 5.05)		1.53 (0.44, 5.41)	
Secondary or Higher	0.21 (0.02, 2.13)	1.25 (0.11, 14.4)	0.56 (0.05, 5.84)		0.69 (0.06, 7.42)	
None Reported	1.18 (0.30, 4.72)	4.01 (0.72, 22.5)	1.48 (0.37, 5.96)		1.60 (0.39, 6.50)	
Asset index quintile	*p* < 0.001	*p* < 0.001	NA	NA	NA	NA
Lowest	1 (ref)	1 (ref)				
Second	1.43 (0.80, 2.57)	1.08 (0.48, 2.47)				
Middle	0.61 (0.26, 1.43)	0.80 (0.31, 2.07)				
Fourth	0.31 (0.13, 0.74)	0.36 (0.15, 0.91)				
Highest	0.12 (0.03, 0.38)	0.15 (0.04, 0.58)				
ANC visits	*p* = 0.009	*p* = 0.006	*p* = 0.010	*p* = 0.006	*p* = 0.051	*p* = 0.015
2 or fewer	1 (ref)	1 (ref)	1 (ref)	1 (ref)	1 (ref)	1
3 visits	0.37 (0.17, 0.82)	0.34 (0.14, 0.81)	0.33 (0.14, 0.80)	0.32 (0.13, 0.27)	0.36 (0.14, 0.89)	0.32 (0.13, 0.78)
4 or more	0.38 (0.20, 0.72)	0.34 (0.16, 0.71)	0.35 (0.17, 0.71)	0.33 (0.17, 0.66)	0.40 (0.18, 0.88)	0.33 (0.15, 0.72)
Relationship to head of household	*p* = 0.506	*p* = 0.866	*p* = 0.646	NA	*p* = 0.579	NA
Self	1 (ref)	1 (ref)	1 (ref)		1 (ref)	
Wife	0.76 (0.33, 1.77)	1.07 (0.38, 3.03)	0.84 (0.36, 1.93)		0.80 (0.34, 1.87)	
Other	0.55 (0.20, 1.51)	0.76 (0.26, 2.20)	0.63 (0.23, 1.71)		0.59 (0.21, 1.62)	

*P-values are based on a Wald joint significance test

^a^For these ORs, 1111 facility births were matched to 59 BBAs on asset index prior to analysis

^b^For these ORs, 876 facility births were matched to 58 BBAs on asset index and age at first pregnancy prior to analysis

#### Matched pairs analysis

The predictor variables included in the matched pairs analysis are presented in Table 2 (Column 1). In the process of identifying key variables for matching, wealth - as represented by asset index quintile - was the strongest predictor of BBA with an adjusted odds ratio of 0.15 when comparing the odds of members in the highest asset quintile experiencing a BBA to those in the lowest quintile, and wealth was significantly associated with every predictor except number of ANC visits. After matching BBAs with facility births on wealth, most bivariate relationships with BBA were weakened, although high parity, younger age at first pregnancy, and low number of ANC visits remained significantly associated with greater odds of a BBA (Table 2, Column 3) and were included in an adjusted, matched analysis. In the adjusted matched analysis (Table 2, Column 4), parity and ANC visits remained significant. Age at first pregnancy was selected as the matching variable for the second round of matching due to the relatively large effect size (adjusted ORs of 0.38 and 0.68 when comparing women in the oldest and second oldest age groups at first pregnancy to the youngest) and its strong relationship to the remaining predictors. In the second round of matching where BBAs were matched with facility births on both wealth and age at first pregnancy, unadjusted odds ratios for all remaining predictors were again estimated (Table 2, Column 5). Only parity and ANC visits were statistically significant and retained for the final model (Table 2, Column 6). Having had a previous birth was associated with an increased risk of BBA (OR > 4 when comparing women with 2 or more total births to women with 1 birth), while attending three or more ANC visits was associated with a reduced risk of BBA (OR ≤ 0.33 for women who attended 3 or more ANC visits compared to those with 2 or fewer) after controlling for wealth and age at first pregnancy. We did not find evidence of an association with age or education.

Lower quintiles of wealth were the strongest predictors of BBA in this study. Higher parity and fewer ANC visits continued to significantly predict higher odds of BBAs once wealth was controlled for via matching. These predictors were associated with age at first pregnancy, matching on age at first pregnancy did not attenuate the relationship between the outcome (BBA) and parity and number of ANC visits.

Taken as a whole, these variables serve as a useful indicator of which women may be at greatest risk for BBA. However, in a context where many women are poor, multiparous, and make multiple ANC visits, their predictive power is limited. It is therefore valuable to qualitatively understand the process by which these and other indicators may inform our understanding of BBAs.

### Qualitative results

The qualitative cases draw from four BBA experiences detailed by Neema, Aisha, Subira and Mwajuma with their husbands Abasi, Jamil and Mosi (see Table 3). Mwajuma’s husband was unavailable for an interview. In all BBA experiences within the qualitative study, all women were married, had experienced at least three previous deliveries and had made multiple ANC visits. Three women reported that at the outset of labor, they began searching for their husbands who were unavailable as they were farming, socializing or running errands; one woman said she was on a bicycle outside her home village when labor began and it was unclear in the interview if she sought her husband. These four BBA deliveries occurred on a bus, in a roadside field, at the home of a stranger and at the home of relative who lived en route to a facility. All four women described in detail their desire to deliver in a facility because they had either experienced a difficult pregnancy, viewed home births or TBAs as “dangerous” or “old fashioned”, or learned from a provider during pregnancy that they were at risk for complications during delivery. Three of the women (and each of their spouses) hesitated at the outset of the interview to state that they delivered en route and instead said they delivered at a facility or at home. Only upon probing in an open-ended format did fuller details of the birth - including its location - emerge. All case study respondents described BBAs in a straightforward, factual manner and, at some point in the interview, described birth as a potentially dangerous fact of life that requires placing life in the hands of God. In each case, women lived relatively far from a health facility (three women lived roughly 15 km from a functioning health facility, while one woman lived three kilometers from a facility). Assessment of geographic access to care is complex, however, as not only distance but also rainfall and river levels, road conditions and transport availability or cost can affect geographic access and travel times. Geographic access was assessed by asking women to describe via distance or time their location relative to a facility. To read longer-form narratives of each case, please refer to Additional file 3.

**Table 3:** Characteristics of qualitative BBA cases (+partner)

Case	Case 1. Neema (+Abasi)	Case 2. Aisha (+Jamil)	Case 3. Subira (+Mosi)	Case 4. Mwajuma
Characteristics				
Maternal				
Parity	4	7	4	4
Age range	35–39	40–44	35–39	25–29
Marital status	Married	Married	Married	Married
Education level	Primary complete	Some primary	Some primary	None
Husband education	Primary complete	None	Some primary	None
Place of previous deliveries	Health facility	Health facility	Disputed	Home
			Subira: Two BBAs; 1 home delivery	
			Mosi: Home	
Distance to facility (km)	≤3	≤15	>15	>15
Pregnancy				
Antenatal visits (#)	2	>4	2–3 (unclear)	4
Referral for delivery	No	Yes	No	No
Birth				
Time between woman’s statement of desire to depart and departure	1–2 h	2–4 h	Disputed	1–2 h
			Subira: left for facility before labor started	
			Mosi: reports home delivery	
Mode of transport to facility	Bicycle	Foot, Bus	Bicycle	Foot
Escort to facility	Husband	Disputed	Disputed	Mother-in-law, Sister
		Aisha: Sister-in-Law	Subira: other pregnant woman	
		Jamil: himself and his sister	Mosi: n/a	
Who was present at birth	Disputed	Disputed	Disputed	Mother-in-law, Sister, Sister-in-Law
	Neema: alone	Aisha: sister-in-law, bus riders	Subira: a pregnant friend	
	Abasi: husband present	Jamil: self, sister-in-law, bus riders	Mosi: their teenage daughter	
Birth location	On the side of a dirt road, near thorny bushes	Public bus	Disputed	Sister-in-law’s house
			Subira: stranger’s house	
			Mosi: own house	
Delivery complications	Mother fell unconscious	Vaginal tearing	None mentioned	None mentioned

The cross-cutting theme or *core consistency* [36] that emerged most strongly across BBAs was the experience of husband-wife discord regarding when and whether to depart at the onset of labor; wives were keen to depart, but their spouses were unreachable or were delayed due to a struggle to prepare financially for the birth (e.g. to procure money for transport, birth equipment or to cover facility fees) or to arrange logistics (e.g. fixing bicycles for departure). A second core consistency involved divergent accounts that emerged in the re-telling of the BBA experience comparing husband and wife narratives.

#### Core consistency 1. Partner negotiation and delays due to economic hardship

Mwajuma, Aisha and Neema each described a need to notify their husbands at the onset of labor pains and to coordinate with husbands in order to reach a facility. This reliance contributed to a substantial delay, which women highlighted in re-telling their stories. Mwajuma (whose husband was at a neighboring brewery) waited in vain for his return, and eventually left for the hospital without him (but only after the decision was sanctioned by his family). Aisha waited at least one hour before she could successfully notify her husband, Jamil, of labor’s onset and then begin walking to a bus stand where she waited several hours for him to arrive with transport funds. Neema tried on at least two occasions to press her husband, Abasi, on the urgency to leave, but was met with his resistance. In each example, an element of the gendered dynamics of decision making for childbirth emerged wherein an activity that involved expense and/or leaving the household required not only permission, but also partner collaboration and financial or logistical scrambling [39]. Husbands shared several reasons for delaying departure. Jamil needed time to request and receive loans from friends, neighbors and family members. Abasi needed to prepare the bicycle for transport and was skeptical of the urgency to leave given the long labors of previous births and a sense that a home-based birth was an equally viable option. Mosi described how poverty constrained every aspect of his and Subira’s lives including decisions on whether to deliver in facilities. Mwajuma’s husband was unavailable for an interview.

Neema and Abasi’s discord regarding when to depart is illuminating in several respects. Neema described frustration with Abasi for not sharing her conviction regarding the superiority of services available in a facility. Careful evaluation (informed by two antenatal visits during this pregnancy and the experiences of three previous, facility-based births) led Neema to prefer biomedical health care. She spoke dismissively of TBAs as “those who should not exist in these modern times.” She described how TBAs lack sterilization equipment, or what Whittaker (1999) termed “technologies of birthing,” and lacking these technologies they represented the antithesis of what she wanted for herself and her baby during birth [40]. By comparison, Neema conferred admiration, power and status on clinical health officers and nurses. Throughout the interview she used the phrase “real medicine” to describe the services they provide. Neema’s husband, however, preferred local practices and knowledge, which he viewed as acceptable, affordable and feasible. He wanted to avoid spending several hours at a facility during his wife’s labor.

Where discord between Neema and Abasi centered on preference for place of delivery based largely on opinions of health care providers, economic constraints proved to be of central importance in delaying departure for Aisha. Aisha awoke in the morning knowing she was in labor, but at least 6 hours passed before she procured funds to board a bus that could take her to a health facility; she eventually delivered aboard the bus. Jamil lamented the memory of his wife standing by the road watching busses pass and being unable to board. He placed blame on himself. “They left her,” he said, “because I hadn’t collected enough money.” Jamil described how as a subsistence farmer and father of six children, his financial status had eroded to a point where he had no savings, “not even a shilling.” Jamil’s feverish, hours-long search for funds and later success securing a loan from a shopkeeper were too late to prevent a birth that was witnessed by many curious bus commuters (to Aisha’s embarrassment), but not by a health professional or TBA. Financial desperation was equally strong in the BBA account of Subira and Mosi. While details of the BBA remained unclear despite follow-up interviews and probing, Mosi consistently described his sadness at his wife’s non-facility delivery. He described feeling “humiliated” at the situation in which his family was living and their inability to access adequate health care. “We came here to look for a better life,” he said, referring to their work as migrant farmers. “I don’t like having to tell you about (this delivery). I feel ashamed. With money, I could send Subira to a (maternal waiting facility), or rent a house and find a person living close to nurses and doctors who could help her deliver… we are so ashamed.”

#### Core consistency 2. Multiple versions of a BBA narrative

At the outset of the interviews, three woman in this study stated that their birth occurred in a health facility, as instructed by providers during ANC visits. Aisha described how during antenatal care, she was referred to deliver in a higher-level facility due to concerns about her age (>40) and parity (7). Mwajuma noted that she needed to deliver in a facility due to anemia, while Subira said her provider discussed her history of pre-term births and the subsequent necessity of facility delivery. Only upon probing for details of their experience did the BBA nature of each birth emerge, which was then coupled with women’s explanations regarding their *intention* to deliver in a facility due to the superiority of biomedicine. Neema was forthright in describing her birth as a birth en route, but clarified this statement by noting that all three of her previous births were in a facility.

Across husband and wife pairs, accounts of BBAs could not be easily reconciled. Jamil first described Aisha’s delivery as occurring at a hospital with him nearby. In a follow-up interview, he described the delivery as occurring aboard a bus in his presence. Aisha first described the birth as occurring in a hospital with a sister-in-law present. In the same interview she revised her account to describe the birth aboard a bus, again with her sister-in-law present. Similar to Jamil and Aisha, Neema did not describe Abasi as being present during the birth, while he described himself as present in both interviews. Subira and Mosi described highly divergent birth accounts wherein Subira described a BBA, which occurred in the house of a stranger while en route to a facility, while Mosi described a home birth.

## Discussion

Drawing on quantitative and qualitative methods, this study explored the experience of BBA among women living in rural areas across Morogoro Region in Tanzania.

A factor that emerged as critically important in both surveys and interviews and which correlates with a wide body of anthropological and epidemiological literature - as well as some BBA literature - was the role of poverty and the ways in which husband-wife pairs of a low socio-economic status are at a disproportionately higher risk of experiencing a BBA [11, 17, 22, 41–43]. In this study, economic constraints amplified discord on delivery location. Although Tanzania's official policy states that delivery services should be free of charge, husbands in this study described concerns related to fees and reservations regarding quality of care, factors that have been described in several studies conducted in Tanzania [42–47]. Partner disagreement on delivery location preference in Tanzania has been significantly associated with reduced rates of facility births; when both partners rated the skills of government doctors and nurses as higher than that of TBAs, women were twice as likely to deliver at a health facility than in the home, even after controlling for confounders including age, wealth, and education [39]. Women’s delayed departure illuminates how, in a context of not only unequal power relations but also severely constrained economic resources, it is largely beyond the control of laboring mothers to determine when they will depart and how they will reach a facility. It is also largely beyond the control of husbands to fulfill their socially-expected breadwinner role and provide funds for transport and birth supplies (such as razors, gloves, a plastic sheet and a new kanga or cloth) [25].

Quantitatively, once wealth was controlled for via matching, higher parity and fewer ANC visits continued to significantly predict higher odds of BBA. The protective effect of multiple ANC visits has been consistently highlighted in both BBA literature [3, 4, 7, 10, 12, 14, 15] and maternal health literature as a means to monitor health, promote health-seeking behavior and devise birth preparedness plans [48]. Our qualitative interviews highlighted potential causes of this relationship: exposure to formal care through ANC visits offered educational opportunities to women regarding facility births and provided women with experiences that increased their sense of trust in and value of facility-based care. The role of parity has also been highlighted in previous BBA studies [2, 3] that offer both biological and behavioral explanations for the relationship of parity to BBA. In the cases presented here, we see women and especially husbands making careseeking decisions based on prior experiences, with one husband describing how he delayed care to avoid the long wait times experienced during previous deliveries. Unlike earlier studies [49], this study did not find trends by educational level.

Multiple birth narratives were elicited during in-depth interviews. Because the research design allowed for follow-up interviews, interviewers sought to probe on divergent accounts of births, particularly in instances where birth accounts appeared contradictory within or across respondents. In the interest of building trust, maintaining confidentiality, and not causing undue strain between husband-wife pairs, extensive probing in the name of triangulation was not undertaken and several inconsistencies remain unexplored. The context of this study involved social spheres and spaces wherein respondents likely placed themselves within a different “social location” [50] than interviewers and may have therefore felt compelled to adjust accounts. This has been referred to in the literature as “a performance of identity” with an understanding that respondents may sometimes engage in presenting “what I think might make me valued by others” or by revealing a “preferred self” rather than an “essential self” [50]. Interviewers – all urban, multi-lingual, college graduates – likely drew upon their own subjectivity during interviews, thereby influencing the production of knowledge as it related to BBA account. In analyzing and presenting these narratives, rather than seeking an objective truth, we sought to engage in a “dialogue with the transcripts, listening to them and asking questions of them” to determine a “contextual truth” [51]. While reading for a contextual truth does not lead to a single, objective truth, we argue that it does illuminate how women and men reshape the story of their lives in an adaptive, socially desirable manner. In viewing the data this way, we recognize potential social pressures women may feel to tell researchers that they delivered in health facilities. We also appreciate the pressure men may feel to present themselves as physically present alongside women throughout labor and delivery, and as financially capable of providing for women before and during childbirth. Given this understanding, we suspect that our survey and other surveys related to careseeking for childbirth, underestimate the number of BBAs and overestimate the number of facility-based births (and potentially also the number of home births).

While it would be impossible to prevent all BBAs, strategies have been proposed to minimize incidence [2]. Interventions could consider re-affirming the importance of birth preparedness plans (with preparedness messaging directed at women *and* their husbands), expanding or improving the capacity of maternal waiting homes, and instructing families during ANC visits on a minimum amount of care required in the event of a BBA (such as keeping a baby warm, cutting a cord with a clean razor and ensuring delivery of a placenta) [7, 12, 18]. While respondents in our study attended several ANC visits where they were instructed on the importance of bringing supplies for birth, participants did not discuss being guided on how they could gradually save funds to afford costs associated with birth, which represents an opportunity for improved birth preparedness messaging.

This study is limited in that it relies on a very small qualitative data set of four women who delivered en route and three of their partners. The study was strengthened by the use of both qualitative and quantitative methods. Qualitative methods highlighted the reality of BBAs to the research team, which informed the decision to include a measure of births en route in the survey. Quantitative measures allowed us to examine generalizable trends in the data and assess statistical trends in light of narrative themes. The uniqueness of this study stems from not only presentation of BBA data in an East African context, but also from the presentation of a male perspective on BBAs. As partners to women and fathers to children, men exert positive and negative influences over maternal health [52]. The influence of men’s intentions and practices on childbirth has been described as “little studied” [52] and in contexts such as Tanzania we urge that more attention be paid toward examining the role of men in careseeking for childbirth. We hope this research sparks more interest in the topic of BBAs and birth preparedness in low-income settings.

## Conclusion

BBAs are understudied globally. In Tanzania, our study found that a low socioeconomic status was the strongest predictor of a BBA. Qualitative interviews highlighted how partner discord regarding when to depart for facilities underpins BBAs. As efforts continue to promote facility birth, we urge policy makers, researchers, clinicians and relevant stakeholders to consider the financial demands associated with facility delivery and the potential for these demands to exclude the poorest of the poor, or place them at a heightened risk for BBAs.

## Additional files

Additional file 1: Quantitative Tool. (PDF 219 kb) Additional file 2: Qualitative Tool. (PDF 117 kb) Additional file 3: Case Studies on Birth Before Arrival. (PDF 95 kb)
