# Supplementary material for: Poverty, partner discord, and divergent accounts; a mixed methods account of births before arrival to health facilities in Morogoro Region, Tanzania
Source: BMC Pregnancy Childbirth. 2016 Sep 27;16:284. doi: 10.1186/s12884-016-1058-x (PMC5039800; doi:10.1186/s12884-016-1058-x)
Supplement: Additional file 1: — Quantitative Tool. (PDF 219 kb) [file 12884_2016_1058_MOESM1_ESM.pdf]

2. Interviewee's personal information.

201.	When were you born? Please tell me the date, month and the year you were born	------------/---------/--------------	
202.	What is your age, in terms of years?<br><br>***COMPARE AND THEN CORRECT 201 AND/OR 202 TO ENSURE CONSISTENCY.***	Write down the age, in terms of years	[ ]
203.	What is your religion?	1. Christian<br>2. Muslim<br>3. Hindu<br>4. Budha<br>5. Others (Mention) ______________	[ ]
204.	What is your tribe?	1. Luguru<br>2. Kaguru<br>3. Pogoro<br>4. Ndamba<br>5. Others (Mention) _____________	[ ]
205.	What activity are you engaged in to earn living?	**Agriculture**<br>1. Crops / Animal keeping<br>2. Fishing<br>**Employed**<br>3.Government and other institutions<br>4.Private sector<br>**Self employed (Not in agriculture/Animal keeping)**<br>5. Has employed other people<br>6. Has not employed anyone<br>7. S/he is involved in family projects (non-agricultural projects) without gaining any payment(s).<br>**Not working**<br>8. S/he is ready to work<br>9. S/he is not ready to work<br>10.Dealing with domestic activities/housewife<br>11. Student<br>12. Can not work (Old age, retired, sick, disabled)<br>13.Other activities<br>(Mention) ___________________________	[ ]
206.	What is your relationship with the head of this household?	1. Head of household<br>2. Wife<br>3. Daughter<br>4. In-law (Married)<br>5. Grand daughter<br>6. Mother<br>7. Mother in-law<br>8. Sister<br>9. Sister in law<br>10. Relative<br>11. Adopted/step daughter<br>12. Domestic servant	[ ]

		13. Friend<br>14. Others-not relatives based: (Mention) _______________	
207.	What is the head of household doing to earn the living? (income generating activity done by the head of household)	**Agriculture**<br>1. Crops / Animal keeping<br>2. Fishing<br>**Employed**<br>3.Government and other parastatals<br>4.Private sector<br>**Self employed (Not in agriculture/Animal keeping)**<br>5. Has employed other people<br>6. Has not employed anyone<br>7. S/he is involved in family projects (non-agricultural projects) without gaining any payment(s).<br>**Not working**<br>8. S/he is ready to work<br>9. S/he is not ready to work<br>10.Dealing with domestic activities/housewife<br>11. Student<br>12. Can not work (Old age, retired, sick, disabled)<br>13.Other activities<br>(Mention) __	[ ]
208.	Have you ever been to school for formal education?	1. Yes<br>2. No ***(GO TO QUESTION 210)***	[ ]
209.	What is your level of eduvation?<br>That is ,how many years did you spend in school or college/university?	Write down the number of years spent in attending school or college/university.	[ ]
210.	Now, i request you to read the following sentence.<br><br>***SHOW THE CARD WITH THAT PARTICULAR SENTENCE TO THAT PARTICIPANT. IF THE PARTICIPANT CAN NOT READ THE WHOLE SENTENCE, INVESTIGATE***: Can you read any part or any words from this sentence?	1. Was able to read the whole sentence<br>2. Was able to read some parts of that sentence<br>3. can not read the given sentence<br>4. Blind/has eye(s) problems	[ ]
211.	Are you currently married or do you live with any man in any marital relationship?	***1. Yes (GO TO QUESTION 213)***<br>2. No	[ ]
212.	**If NO,** what type of marital relationship are you currently in? Widow, divorced, separated from your partner?	1. Widow<br>2. Divorced<br>3. Separated from my partner<br>**4. Never been married – GO TO QUESTION 215**	[ ]
213.	Has your husband or your partner got formal school education?	1. Yes<br>2. No<br>9. I don't know/I don't remember	[ ]
214.	What is the education level of your husband or partner?	Mention the years spent in school	[ ]

215.	How old were you when you became pregnant for the first time?	Years ____	[ ]
**Please let me ask you few questions concerning your household.**			
216.	What is the main source of drinking water and water for other uses in your household?	1. Tap water,, available inside the house<br>2. Tap water, available outside the house<br>3. Tap water, from the community water supply.<br>4. Open water well, found outside the house<br>5. Open well for community uses<br>7. Constructed water well, located outside the house<br>8.Springs/Rivers/water channels/pond/lake<br>9. Other (Mention)___________________	[ ]
217.	Is your household with the toilet?	1.Yes<br>2.No ***(GO TO QUESTON 219)***	[ ]
218.	If yes, what type of toilet is it?	1. Toilet not using water to flash waste materials (not shared one).<br>2. Toilet using water to flash waste materials (shared with other household(s))<br>3. Pit latrine,allowing air in but not shared with other household(s).<br>4. Pit latrine, allowing air in and shared with other households.<br>5. Toilet constructed under traditional settings but not shared with other households<br>6. Toilet constructed under traditional settings and shared with other households<br>7. other type(s) (Mention)________________	[ ]
219.	What are the materials used to roof the house where the head of this household lives in?<br><br>**(INVESTIGATE AND KEEP THE RECORD OF THINGS YOU WILL SEE)**	1. Grasses/ Leaves/ Soil<br>2. Roofing iron sheets<br>3. Tiles<br>4. Cement<br>5. Asbestos<br>6. Another type (Mention)_________________________	[ ]
220.	What is are the materials used to make the walls of that house?<br><br>***(KEEP THE RECORD OF THE THINGS YOU WILL SEE. IN CASE MORE THAN ONE MATERIAL HAS BEEN USED, SHOW THAT ONE THAT HAS BEEN USED MAINLY)***	1. Trees and soil<br>2. building blocks made using soil<br>3. building blocks burnt in furnaces<br>4. Timber<br>5. Cement blocks<br>6. Stones<br>7. other materials (mention) __________	[ ]
221.	What are materials used in making the floor of that house?<br>**(INVESTIGATE AND KEEP THE RECORD OF THE THINGS YOU WILL SEE)**	1. Soil/Sand<br>2. Animal secretions<br>3. Wood planks/bamboo trees<br>4. Polished timber<br>5. Ceramic	[ ]

		6. floor tiles<br>7. Cement<br>8. Other (Mention) _____________	
222.	Is the house mentioned above owned by your household?	1.Yes ***(GO TO QUESTION 224)***<br>2. No	[ ]
223.	If this household doesn't own this house, have you rented it?	1.we have rented the house<br>2. another arrangement (Mention) ___________	[ ]
224.	Does your household own land?	1.Yes<br>2. No ***(GO TO QESTION 226)***	[ ]
225.	If your household owns the land, what is the size in acres ?	Write down(an estimation) the number of acres<br>998 I don't know	[ ]
226.	Is your house with electric power?	1.Yes<br>2.No	[ ]
227.	Is your house with the following?(put a mark on the corresponding item)<br>A. Radio and music instruments<br>B. chickens or ducks<br>C. Cow or donkey<br>D. Sheep, goat, pig<br>E. Bicycle<br>F. motorcycle<br>G. car or tractor<br>H. Television<br>I. Fridge<br>J. mobile phone<br>K. Home telephone (not mobile phone)<br>L. shop/kiosk/bar<br>M. sawing machine<br>N. Sofa/Sofa set.<br>O. Special table for food.	A. 1=YES 2=NO<br>B. 1= YES 2=NO<br>C. 1= YES 2=NO<br>D. 1= YES 2=NO<br>E. 1= YES 2=NO<br>F. 1= YES 2=NO<br>G. 1= YES 2=NO<br>H. 1= YES 2=NO<br>I. 1= YES 2=NO<br>J. 1= YES 2=NO<br>K. 1= YES 2=NO<br>L. 1=YES 2=NO<br>M. 1=YES 2=NO<br>N. 1=YES 2=NO<br>O.1=YES 2=NO	[ ]<br>[ ]<br>[ ]<br>[ ]<br>[ ]<br>[ ]<br>[ ]<br>[ ]<br>[ ]<br>[ ]<br>[ ]<br>[ ]<br>[ ]<br>[ ]<br>[ ]

3 Pregancy history and services received

May i please ask you about your history of prenancy and health services?

301.	Is there any biological child of yours that you are living with currently?	1.Yes<br>2.No	[ ]
302.	How many sons of yours are you currently living with?<br>How many daughters of yours are you currently living with?	***IF NO CHILDREN WRITE '00'***	[ ]<br>[ ]
303.	Do you have any biological children that you are not living with at this	1.Yes<br>2. No **(Go to 305)**	[ ]

	moment?		
304.	If yes, how many sons that you are not living with currently?<br>How many daughters that you are not living with at this time?	The number of male children living far<br>The number of female children living far.<br><br>***IF NO CHILDREN LIVING FAR AWAY, WRITE '00'***	[ ]<br>[ ]<br>[ ]
305.	***REFER QUESTIONS NUMBER: (302 + 304), COMPARE AND THEN CORRECT TO CONFIRM THE REAL TOTAL NUMBER OF CHILDREN***<br><br>Currently you have________living children.	1.yes<br>2 .No	[ ]
306.	Have you ever given birth to a living child who died later?<br><br>***IF THE ANSWER IS NO PROBE: ANY CHILD WHO CRIED OR SHOWED SIGNS OF LIFE IMMEDIATELY AFTER BIRTH BUT DIED AFTER HOURS OR FEW DAYS.***	1. Yes<br>2.No (**Go to question 308**)	[ ]
307.	How many male children of yours have died so far?<br>How many female children of yours have died so far?	***IF NO CHILDREN HAVE DIED, WRITE '00'***	M[ ]<br><br>F[ ]
308.	Some pregnancies could face various intra utero damages or abortions. Some could cause a woman to deliver prematurely or to give birth to a dead baby who has no any signs of life.<br><br>Have you ever faced any of the above situations at different moments of your pregnancies such as giving birth to a dead child?	1.Yes<br><br>2 .No **(Go to question 310)**	[ ]
309.	How many times have these incidences of abortions or giving birth prematurely have happened to you?	Number of such incidences ______	[ ]
310.	Add answers from questions 305, 307, and 309 so that you get the total number of pregnancies and then write on the space given on the right side.	Total number of pregnancies	[ ]
311.	***REFER TO QUESTIONS: (305 + 307 + 309) AND COMPARE AND CORRECT TO CONFIRM THE INTERVIEW ON THE NUMBER OF CHILDREN.***<br><br>For now you have _______children (Question number 305)<br>a number children who died ______	1.Yes<br>2 .No	[ ]

	(Question 307)<br>number of pregnancies delivered prematurely ______ (Question 309)<br>Is given information above correct ?		

**Please tell me about your last pregnancy**			
312.	How many months did your last pregnancy last?<br><br>***WROTE DOWN IN TERMS OF COMPLETE MONTHS.***	**Write down the pregnancny age ___________**<br>***(iF IT IS LESS THAN 7 MONTHS ,GO TO QUESTION 318;OTHERWISE ASK THE NEXT QUESTION)***	[ ]
313.	How many children did you deliver during your last pregnancy	Write the total number of children_________________	
314.	Did that child/children cry, get any shaking of the body or breath after birth?	Child 1 1= Yes, 2 = No 9 = I don't remember<br>child 2 1= Yes, 2 = No 9 = I don't remember<br>child 3 1= Yes, 2 = No 9 = I don't remember	[ ]<br>[ ]<br>[ ]
315.	Is that child/children still alive?	Child 1 1= yes, 2 = no 9 = I don't remember<br>child 2 1= yes, 2 = no 9 = I don't remember<br>child 3 1= yes, 2 = no 9 = I don't remember<br>**IF NO GO TO QUESTION 317**	[ ]<br>[ ]<br>[ ]
316.	If s/he or they are there alive: (NAME) what is her/his age?	Child 1: Months[ ] days [ ]<br>child 2: Months[ ] days[ ] total numbe of days<br>child 3: Months[ ] days[ ]<br>**Go to question 318**	[ ]<br>[ ]<br>[ ]
317.	Did you at any time see a health expert so that he/she investigates about your health when you were pregnant	1. Yes ***(GO TO QUESTION 320)***<br>2. No<br>9. s/he has refused to respond	[ ]
319.	If you met the health expert for investigations into your heath when you were pregnant, where did you get him/her?<br><br>1=YES, HE/SHE MENTIONED<br>2=NO/SHE DID NOT MENTION	A. Morogoro regional hospital<br>B. another hospital_____________________<br>C. health center_________________________<br>D. dispensary _________________________<br>E. pharmacy _____________________<br>F. traditional birth attendant<br>G. other heatlh services centers, mention______________________________	[ ]<br>[ ]<br>[ ]<br>[ ]<br>[ ]<br>[ ]<br>[ ]
320.	If you went to the anti natal clinic for investigations into your health, at least once, please answer the following questions about the investigation(s) that was done to you when you were	A. Was you weight measured?<br>B. Was your height measured?<br>C. Was your blood taken for investigations?<br>D. Was your blood pressure measured/taken?<br>E. Were urine investigations done?	[ ]<br>[ ]<br>[ ]<br>[ ]<br>[ ]

	pregnant<br><br>READ THE ANSWERS, WRITE<br>1=YES<br>2=NO	F. Was your abdomen checked by palpation?<br>G. Were you told of any signs of danger throughout the period of your pregnancy?<br>H. Were you told were to go, in case of any health problem throughout the period of your pregancy ?<br>I. Were you given counselling with regard to HIV testing ?<br>J. Did you have discussions on the mother to child transmission of HIV ?<br>K. Were you given counselling regarding family planning ?<br>L. Were you given mosquito net discount card?<br>M. Did you have discussions on the preparations for delivery?<br>N. Did you have discussions on breastfeeding of the baby continually without mixing any other food?	[ ]<br>[ ]<br>[ ]<br>[ ]<br>[ ]<br>[ ]<br>[ ]<br>[ ]<br>[ ]
321.	How old was your pregnancy when you went to the clinic for first time?	Months__________________________	[ ]
322.	How old was your pregnancy when you went to the clinic for the last time?	Months_________________________	[ ]
323.	How many time did you attend ante natal clinic throughout that pregnancy?	Total number of visits to the clinic _________<br>99= she doen't know<br>**IF IT IS MORE THAN 3 TIMES, GO TO 327**	[ ]

4 Health problems during delivery/intra-utero fetal death(s)

Now i request to ask you about the problems you faced during pregnancies

401	Where was the delivery of your baby conducted?<br><br>***IF SHE DOES NOT KNOW WEATHER IT WAS THE HOSPITAL, HEALTH CENTRE, OR GOVERNMENT CLINIC OR PRIVATE ONE: WRITE DOWN THE NAME OF WHERE SHE IS.***	1. Morogoro regiona hospital **(GO TO 403)**<br>2. Other hospitals (Mention)_____________________ **(GO TO 403)**<br>3. Health center (Mention)_________ **(GO TO 403)**<br>4. Dispensary (mention)_____________ **(GO TO 403)**<br>5. Pharmacy **(NENDA 403)**<br>6. Anther centre for health services (Mention)______ **(GO TO 403)**<br>7. At the home of the traditional birth attendant<br>8. At my home?<br>9. At the home of the community health worker<br>10. At the home of somebody else<br>11. Along the way	[ ]

402	If you did not deliver at the health centre, what was the reason(s)?<br>**1= she has mentioned; 2=she has not mentioned**<br><br>***(don't read out the answers but probe)***	A. A distance and high cost for transport<br>B. time for services does not correspond with schudule<br>C. Unpleasant behavior shown by health provider<br>D. Inappropriate attitude of the health care provider<br>E. Lack of confidentiality<br>F. Lack of medications<br>G. Long waiting time<br>H. High costs of the required service(s)<br>I. Religious reason(s)i<br>J. There is no any benefit(s)<br>K. I did not know the importance of doing so<br>L. I did not the permission to do so<br>N. any other reasons (mention)	[ ]<br>[ ]<br>[ ]<br>[ ]<br>[ ]<br>[ ]<br>[ ]<br>[ ]<br>[ ]<br>[ ]<br>[ ]<br>[ ]<br><br>[ ]<br>[ ]
403	Who helped you at the time of delivery?<br><br>***(DO NOT READ OUT THE ANSWERS BUT PROBE OR ASK: OTHER REASON(S)?<br>1= SHE MENTIONED 2= SHE DID NOT MENTION)***	A. Heath worker at the dispensary<br>B. Health worker at the health center<br>C. Health worker in the hospital<br>D. Health worker in the village<br>E. Traditional healer<br>F. Traditional birth attendant<br>G. Brother/sister/friend/neighbour<br>H. Another one<br>I. She doesn't know/she doesn't remember	[ ]<br>[ ]<br>[ ]<br>[ ]<br>[ ]<br>[ ]<br>[ ]<br>[ ]<br>[ ]

**5 Newborn health<br>Now,i would like to ask you the questions concerning things that were done immediately after delivery**			
501.	Was the baby/babies born with bigger size, bigger size than normal, average size, smaller size than average, a very small size?<br><br>***1. BIGGER SIZE<br>2. BIGGER SIZE THAN NORMAL<br>3. AVERAGE SIZE<br>4. SMALLER SIZE THAN AVERAGE<br>5. A VERY SMALL SIZE THAN NORMAL<br>9. I DON'T KNOW***	Baby 1<br>Baby 2<br>Baby 3	[ ]<br>[ ]<br>[ ]
502.	Was that baby/those babies' (Name/Names) weights measured after delivery.	1=yes<br>2= no (**GO TO 504**)<br>9= she cant say/She does not know	[ ]

503.	If yes, what was the baby/each baby's weight?<br>***WRITE DOWN THE WEIGHT IN KILOGRAMS AS SHOWN IN THE CLINIC CARDS OF THOSE BABIES , IF THEY WILL BE AVAILABLE***	baby 1_______________kg<br><br>baby 2_______________kg<br><br>baby 3_______________kg<br><br>9.	[ ]<br>[ ]<br>[ ]
504.	If the baby was born before time (premature), or if was born with low birth weight, were you give any advice?	1. Yes<br>2. No<br>**3.** The bay was not born before normal time /did not have low birth weight.**GO TO 506**	[ ]
505.	What advice were you given?<br><br>**DON'T READ OUT ALL ANSWERS FOR HER**<br><br>**1=SHE HAS MENTIONED**<br>**2=SHE HAS NOT MENTIONED**	A. Give warmth to the baby by resting him/her on your chest so that you touch each other through skin contacts (Kangaroo Mother Care).<br>B. The baby should be dressed in hut and socks.<br>C. Breastfeed the baby regularly, after every 2-3 hours during the day and night<br>D. If the baby still can not suck the milk well for a long time, you can milk the breast/squeeze out your own milk using a cup to make sure that the baby gets that first yellowish milk.<br>E. Breast milk is the best food among all foods to ensure the baby gains good weight.	A.[ ]<br><br>B.[ ]<br>C.[ ]<br>D.[ ]<br><br>E.[ ]
506.	What was the first thing to be done to the newborn (or the first born) immediately after birth?<br><br>**PROBE DEEPLY ESPECAILLYT IN CASE OF TWINS**<br><br>**ENCYLE ONE ANSWER**	1. Cutting the cord<br>2. To place the baby on the mother's chest/mother's abdomen.<br>3. Baby was left alone.<br>4. Baby was cleaned using a piece of cloth (in order to dry the some water/moisture))<br>5. Baby was covered using clothes<br>6. Baby was bathed<br>7. Baby was left to sleep<br>8. Baby was breastfed<br>9. Baby was given water with sugar or any other thing to eat/drink<br>10. 98=other thing (mention) ______________<br>11. 99=she doesn't know/ she doesn't remember.	[ ]
507.	Was the baby dried up in order to remove the watery moisture from his/her body?	1=Yes<br>2=No ***(GO TO 509)***	[ ]
508.	If yes, was the baby dried up before or after coming out of the placenta?	4 Before coming out of the placenta<br>5 After coming out of the placenta<br>9. She doesn't know/she doesn't remember.	[ ]
509.	Was the baby covered with clothes after delivery	1=Yes<br>2= No ***(NENDA 511)***<br>9= she doesn't know/she doesn't remember **(Go to 511**)	[ ]

510.	If yes, was the baby covered wit clothes before or after the coming out of placenta?	1. 1. Before coming out of the placenta<br>2. After coming out of the placenta<br>9. She doesn't know/she doesn't remember	[ ]
511.	Did your baby cry spontaneously after delivery?	1=Yes **GO TO 514)**<br>2= No	[ ]
512.	If the baby didn't cry immediately, was there anything that was done to the baby to make him/her cry/breath after delivery?	1=Yes<br>2= No **GO TO 514)**	[ ]
513.	If yes, what was done to make the baby cry or breath?	______________________________	[ ]
514.	What was the time duration post delivery when the baby was breastfed?	minutes ______________<br>hours________________<br>days________________<br>***(write down the total number of minutes)***<br>I don't know= 00	
515.	Was the baby breastfed on the initial mother's yellowish milk?	1=Yes<br>2= No<br>9= she cant say/she doesn't know	[ ]

**Newborn babies' health problems**			
524	Did your baby get sick within a month of delvery?	1=Yes<br>2= No(**NENDA 601**)<br>9= He/she does not know.(**NENDA 601**)	[ ]
525	If yes,did the community health worker advise you to go to health facility to get proper treatment?	1=Yes<br>2= No	
526	If a baby got sick within a month of delivery,what was the sickness?<br><br>***(DO NOT READ OUT ANSWERS.PROBE FOR MORE ANSWERS.<br>ASK:OTHER?<br>MARK<br>1= MENTIONED 2= NOT MENTIONED***	A. Baby could not suck the mother's milk.<br>B. Diarrhoea<br>C. Pus in the navel<br>D. A navel becomes reddish.<br>E. Eyes were reddening<br>F. High fever<br>G.<br>H. Lowering of the baby's body temperature<br>I. convulsion<br>J. Burning-like skin effect<br>K. Failure to breath or to cry<br>L. Fast breathing<br>M. Difficulty in breathing<br>N. Failure to urinate<br>O. Failure to defecate<br>P. Cough and flue<br>98.Other(Mention) ________________	[ ]<br>[ ]<br>[ ]<br>[ ]<br>[ ]<br>[ ]<br>[ ]<br>[ ]<br>[ ]<br>[ ]<br>[ ]<br>[ ]<br>[ ]<br>[ ]<br>[ ]<br>[ ]<br>[ ]
