# Supplementary material for: Poverty, partner discord, and divergent accounts; a mixed methods account of births before arrival to health facilities in Morogoro Region, Tanzania
Source: BMC Pregnancy Childbirth. 2016 Sep 27;16:284. doi: 10.1186/s12884-016-1058-x (PMC5039800; doi:10.1186/s12884-016-1058-x)
Supplement: Additional file 3: — Case Studies on Birth Before Arrival. (PDF 95 kb) [file 12884_2016_1058_MOESM3_ESM.pdf]

Case Study 1. Neema & Abasi Mvomero District

Neema has delivered 3 children and experienced 1 miscarriage. Neema's newborn daughter Pili was delivered among thorny bushes by the side of a road. This marked the first non-facility birth for Neema, who views home births and traditional birth attendants as outdated. Neema's husband, Abasi played a critical role in both seeking emergency care en route but also delaying Neema's time to departure for a facility. Neema lost consciousness during her delivery, which occurred while she was alone.

Four months ago, Neema woke early, grabbed her hoe and headed to the farm. Upon arrival, she felt a tight cramping at her waist and returned home to resume less strenuous work. As the cramping built, she grew suspicious, "I thought to myself, these are labor pains." This was confirmed when she went to relieve herself and discovered spotting. She began packing a bag of her nicest clothes – clothes that she described as fit for facility delivery – and awaited the return of her husband who was running an errand. Upon his return, she told him they needed to start walking to the facility. He dismissed this, urging her to relax and recall how long her previous labors lasted. "It's true," she said, "with all my boys labor lasted upwards of 9 hours." Nevertheless, this time something felt different and Neema was concerned. The thought of delivering at home, while reasonable to Abasi, was anathema to Neema. She had never delivered a child at home and dismissed those who did. "TBAs should not exist in these modern times. I don't trust them. We're told not to go to them. The same instruments that they use on you, they could have used on 10 other women. For them, sterilization is just laying instruments in the sun. I prefer the facility." Feeling her cramps coming stronger and faster, Neema insisted that she needed to leave now. But Abasi insisted that he needed to run errands (related to departing for the facility and to household needs generally). He first went to a repairman to fix their bicycle. He then greeted a friend at a nearby shop. "Without him," Neema said, referring to her husband. "I may have made it on time." At around 8:30 am – 1-2 hours after she asked that they depart – Abasi grabbed his bicycle, tied her bag of clothes to it, eased her onto a seat and began peddling to the facility. Not a quarter mile into the 2-mile journey, Neema was overcome with pain and told Abasi to stop. "I told him, 'I'm having real difficulty here.' We were right there on the road, just the two of us and thorns everywhere. But what can we do?" Abasi stopped and helped ease Neema onto the sandy ground. He was nervous. "We were alone in the bush," he said. He felt ill-prepared in case the birth was happening and asked Neema if he should fetch some women he had seen farming a few fields back. Neema agreed to this plan. In his absence, to Neema's surprise, her water broke. With her previous children, her water broke and hours later she delivered. This time her water broke and she felt a desperate need to push. "I was alone, by myself. I laid back, grabbed my leg and started pushing," she said. In the midst of the delivery, Neema began feeling faint, like she needed to "doze off". She felt like she was coming in and out of a sleep when she briefly awoke and saw a baby lying on the ground near her feet. She remembered thinking that the baby looked cold, "So I covered her in cloth, but I didn't have the strength to hold her." Instead, she laid the wrapped baby on the sand and fell back asleep. Some time later (she was unsure of how long) she snapped back into consciousness, saw a baby girl on the ground and pulled her close to her chest.

In her recollection, Neema delivered alone. In Abasi's account, he says he was with her at the delivery. Neema recalls that Abasi returned with a group of women post-delivery as she was rubbing sand and dust off of herself and her baby. The women scolded her and said, "You have rested enough. Now get up and go to the facility." As a response, Neema unfolded the cloth to reveal her newborn who was "looking healthy, already sucking on her fingers". When the women realized that she had already delivered, they ordered Abasi to leave in search of a TBA. In Abasi's memory, there was already an attendant within the group. Neema said the placenta had not yet delivered and nobody was sure what to do. Within minutes, Abasi returned with a TBA who had a razor blade. She immediately cut the umbilical cord, wrapped the newborn and handed her to a woman to hold. She then instructed Neema, who was standing, to crouch and squeeze (*mbavi*) while she massaged and pressed her in order to deliver the placenta. The placenta came out roughly 30 minutes after the delivery, within moments of the TBA's massaging. About two hours after the delivery, the TBA advised Neema and Abasi to resume their journey to the facility, but Neema declined. "My stomach was hurting a lot and the distance felt very far." The baby appeared healthy to all and Neema and Abasi returned home. They registered and immunized the baby the following day, and a provider scolded Neema for delivering on the way. "They told me, 'Why did you not deliver here at the facility?' and I said, 'Because God brought me a blessing while on the way.'"

Case Study 2. Aisha & Jamil in Kilosa District

Aisha has delivered 7 children, 6 of whom are living and 1 who has died. Aisha's newborn daughter Zaina was delivered among numerous onlookers on a crowded bus en route to a referral facility. In the past, Aisha delivered all of her children at a facility. Because of Aisha's age and parity, providers at her closest health center referred her to the larger district hospital during pregnancy. Aisha's husband, Jamil, struggled to find funds to cover transport to the hospital and to purchase supplies required for facility deliveries, including a basin and gloves. In a first round of interviews, Jamil described Aisha's delivery as a routine event that occurred in a health facility. Upon follow-up interviews, he elaborated on the birth aboard a bus. While Jamil places himself at the delivery, Aisha said she was escorted only by her sister-in-law. Aisha tried to stall the delivery of her baby; she suffered extensive blood loss and vaginal tearing.

Aisha recalls awaking one recent morning with labor pains. As a self-described "mature mother", she knew these marked the start of labor, thoughts that were confirmed when she noticed spotting. At around 7 am, she sent a child to fetch her husband, Jamil, at their farm. When her husband returned, he told her they had no money and he would need to seek funds from friends and neighbors. He was already in debt to several neighbors. Aisha decided to get a head start on the journey, accompanied by a sister-in-law, with an expectation that her husband could catch up. After 2-3 hours of walking, they arrived at a town that had a routine inter-village bus. At around 11 am, Aisha watched as first a bus and later a car drove past en route to the town where her hospital was located. Without any money, she couldn't board either vehicle. "They left her standing there," her husband Jamil said with regret, "because I hadn't collected any money." When her husband arrived to the bus station, around noon, he was empty-handed. "So then he was there with me, but where could he borrow money right there on the road? He went here and there, but they were all refusing him." Aisha began feeling desperate. "I was praying to God, praying to God," she said. Jamil described a frustrating search for funds: "I just did not have money. None at all, not even a shilling. I had to go around. I went down there to that guy and asked. Then I found another guy. I found the chairman of the village government. Finally, I found a guy who lives down that way… he called another guy who is a petrol vendor and that young man gave me 30,000 shillings (~$18). I gave it to my wife and we left for Kilosa." The next bus came at 2pm. While Jamil says that he traveled with her to the facility, Aisha says that she boarded with only her sister-in-law. Once aboard, Aisha was "overwhelmed (*kabiliwa*)" and "struggling, really struggling." She had wanted to either get off the bus or to ask the driver to go directly, without stopping, to the final city where her hospital was located. "An ambulance that carries sick people would go straight to a hospital. But this is a bus that has to pick up passengers. So you can't say, 'Please rush me to the hospital.' They go according to their own schedule. "At the bus' terminal point, as passengers began disembarking, Aisha could not stand. Once the passenger sitting next to her stood, Aisha felt her lower body tighten. Her legs grew heavy and tight. Her escort urged Aisha to try to disembark, but "I could not. I just could not come out." Instead, Aisha re-positioned herself to lay prone on the bus bench. Despite efforts to delay the birth, Aisha's daughter "came into the world with force." Her sister-in-law, a teenager, caught the baby while men, women and children stared through windows and peered over nearby bus seats. Aisha tried to cover her baby and herself with her dress. The driver repeated that all passengers must disembark so that he could drive directly to the facility. Upon arrival at the hospital, around 4 pm, the conductor ran into the facility to alert providers that a delivery occurred on his bus. Two nurses rushed out; one cut the umbilical cord and wrapped the baby while another delivered the placenta, which was expelled around 30 minutes after delivery. Aisha praised the providers. "Those nurses did not shout at me, they didn't tell me that I was old nor did they ask me about delivering at home…They held me by the hand and helped me get into the ward," she said. Once in the ward, providers began stitching tears in Aisha's vaginal wall. "I was surprised they were so bad because I couldn't feel it," she said. As Aisha and Jamil describe it, transport costs were a major barrier leading to BBA. "If I had money and got on the 11am bus, I would have delivered in the [hospital labor] ward. I did not get on the first bus, I had to get on the later bus." Aisha laughed while summing up her experience. "So that's how it was on the day of fools," she said. "April 1st is when I delivered."

Case Study 3. Subira & Mosi in Ulanga District

Subira has delivered 4 children. She and her husband Mosi presented divergent accounts of her birth experience with their daughter, who was born 2 weeks earlier. Mosi said the child was delivered at home; Subira said the child was delivered on the way. Husband and wife agreed that Subira experienced at least 1 BBA several years ago as well as at least 2 home deliveries. The couple also agreed that the journey from their village to a facility takes 4-5 hours by bicycle. Motorcycles are prohibitively expensive at 30-40,000 shillings (~$18-24 USD). As recent migrants to the region they felt heightened uncertainty about how and where to access care.

Subira said that at around 7am two weeks ago, she and several friends who were also pregnant began bicycling to a facility to attend a routine antenatal care visit. One hour into the journey, she felt a jolt of pain and began peddling faster, fearing that this could be labor pain. Eventually she could no longer peddle. She tried to sit on the back of her friend's bicycle, but her legs started buckling in pain and she had to get off the bicycle. By that time, she was in a neighboring village. Her friends escorted her to a stranger's house, explained that she had a "stomach ache", and she was given a room where she delivered with her friend's assistance at 9am. It is unclear who cut the umbilical cord and what supplies were used. The placenta came out immediately following the delivery. She rested for an uncertain amount of time, then hired a motorcycle for 5,000 shillings to bring her, her baby and her friends back to the village. While there is a maternal waiting home near the facility she was intending to reach, she could not access it for two reasons. First, she did not know her delivery date. Second, she did not want to leave her children at home with their father. "You know men," she said. "The children cannot stay with him. What would they eat?" When probed on risks associated with a father watching over his children versus a mother delivering on the way, she said, "Death can come anytime. Death can even come in a facility."

Mosi recalled that at around 3am Subira began feeling labor pain, and because neither she nor Mosi knew how to get her to a facility, she gave birth in their home at around 9 am aided by their adolescent daughter who had delivered three of Subira's previous children and who Subira instructed throughout the delivery process by explaining how to catch the baby and how to cut the umbilical cord using a new razor. Her daughter was not wearing gloves. Mosi said he was standing outside, praying to God and listening to his wife during the delivery. He described feeling "humiliated" at the situation he and his family live in, as it results in experiences such as home deliveries." We came here to look for better life…I don't like having to tell you about (this delivery). I feel ashamed. With money, I could send Subira to a (maternal waiting facility), or rent a house and find a person living close to nurses and doctors who could help her deliver. But we are so ashamed."

Mosi and Subira both said the baby has not been taken to a facility since birth, but Subira said a vaccine truck passed through on the day of the interview and a nurse vaccinated and weighed the baby. When Subira explained the BBA to the nurse, she thought she may get yelled at for being unprepared. Instead, the nurse comforted her and said, "This was God's plan."

Case Study 4. Mwajuma in Ulanga District

Mwajuma has delivered 4 children. Mwajuma's daughter Neema was delivered in the presence of her mother, sister and sister-in-law in her sister-in-law's house hours before the interview. In the past, Mwajuma delivered her children at home. Her recent experiences with anemia coupled with repeated warnings from her providers regarding home delivery made Mwajuma feel determined to deliver in a facility. During the middle of the night, while en route to the facility, Mwajuma intended to briefly stop in a village where her sister-in-law lived and her husband was socializing. While there, she delivered. She was still recovering from the birth at the time of this interview.

In previous births, Mwajuma felt comfortable delivering at home. But this time, throughout her pregnancy, she felt weak and providers told her that due to risks associated with anemia a facility delivery was necessary. Mwajuma was determined to deliver at a facility despite living several hours from the nearest health center. The day before this interview at around 4 or 5 pm, she began to feel intense cramping. She waited for her husband to return home, in order for them to depart together. Later, as it appeared that he may not return, waiting felt impractical. Mwajuma decided (following consent from her mother-in-law and sister-in-law) to begin the journey, and hoped to find her husband en route. During this 1-hour walk, they had to stop several times, but eventually they reached a village brewery where her husband was drinking. This was still several miles from the facility and it was dark outside. Mwajuma and her family decided to rest at a sister-in-law's house, in the same village. Mwajuma waited while her mother-in-law gathered supplies necessary for deliveries including gloves, a plastic sheet and a clean razor. It is unclear where Mwajuma's husband was at this time. As Mwajuma's cramps began to intensify, around midnight, her sister decided to seek out a local TBA who could either serve as an escort in the morning or help with a home delivery. Soon after the arrival of the TBA, Mwajuma's mother-in-law laid out a sheet of plastic and the baby was born. The TBA wore gloves. She tied the umbilical cord with a thread of dress fabric ("kanga") before Mwajuma's mother-in-law cut the cord with a new, 100 shilling ($0.05) razor. The placenta was expelled almost immediately after delivery. Mother and baby were washed after delivery. Mwajuma insisted several times during the interview that she "wanted to deliver in a facility" particularly as evidenced by her frequent facility visits but "the cramps were strong and fast" and eventually, she simply "could not walk." Sometime in the near future she said she will take her daughter to a facility, where she expects to receive good care despite some concerns that she may be reprimanded for not delivering in a facility.
